# Strengthening disaster preparedness and health security in Niger state, Nigeria through a WHO STAR–based multi-hazard risk assessment

**DOI:** 10.1038/s41598-025-34702-z

**Published:** 2026-01-16

**Authors:** Oladayo David Awoyale, Akolade Jimoh, Anne Dede, Catherine Nabiem Akpen, Abiodun Ogunniyi, Dennis Paul Dogo, Patrick B. Gimba, Idris Ibrahim, Rauf Rauf, Arab Mustafa, Grace Erekosima, Nimatullah Ibrahim, Sunday Atobatele, Sidney Sampson, Hilary I. Okagbue

**Affiliations:** 1 Sydani Initiative for International Development, Sydani Group, Abuja, Nigeria; 2 Sydani Institute for Research and Innovation, Sydani Group, Abuja, Nigeria; 3 Nigeria Centre for Disease Control and Prevention, Abuja, Nigeria; 4 Ministry of Secondary and Tertiary Health, Minna, Niger State Nigeria; 5 Department of Mathematics, Covenant University, Ota, Nigeria

**Keywords:** Disaster risk reduction, Preparedness, Hazards, WHO STAR, Niger state, Diseases, Environmental sciences, Health care, Natural hazards, Risk factors

## Abstract

Niger State in central Nigeria faces a range of natural, biological, and security hazards. To inform preparedness and health security planning, a multi-hazard risk assessment was conducted using WHO’s Strategic Tool for Assessing Risks (STAR), this is one of the first applications of WHO STAR at a state level in Nigeria. A cross-sectional study was conducted using the WHO STAR. Stakeholders involved identified hazards across natural, biological, technological, and societal domains through review of surveillance, disaster, and meteorological data. Hazards were scored for likelihood, impact, vulnerability, and coping capacity, with composite risk indices used to rank and categorize them. Priority hazards were further analysed for seasonality and geographic distribution, and findings validated through consensus. Eighteen major hazards were identified, spanning biological, environmental, and societal. Seven hazards emerged as very high risk, notably flooding, banditry/kidnapping. Six were high risk (e.g. fire outbreaks), four moderate (e.g. acute flaccid paralysis), and one low risk (diphtheria). Six hazards showed clear seasonal patterns. Priority hazards were further examined for geographic distribution and validated through consensus. The STAR assessment produced an evidence-based risk profile highlighting flooding, banditry/kidnapping, boat mishaps, cholera, and rain/windstorms as the most critical hazards. Actionable recommendations were developed to support preparedness, mitigation, and response efforts across sectors. The findings offer a structured basis for strengthening disaster risk governance and can inform the development and implementation of Niger state’s emergency preparedness plans.

**Supplementary Information:**

The online version contains supplementary material available at 10.1038/s41598-025-34702-z.

## Introduction

Disaster risk reduction (DRR) is a key component of sustainable development and global health security, which seeks to reduce the negative impacts of hazards on people, infrastructure, and economies^1^. Global frameworks, such as the Sendai Framework for Disaster Risk Reduction 2015–2030, highlight four priorities: understanding disaster risk, strengthening disaster risk governance, investing in disaster risk reduction for resilience, and enhancing disaster preparedness for effective response and “build back better” in recovery, rehabilitation, and reconstruction^2^. These priorities are interrelated, and their success depends on governments and communities’ abilities to identify and address the core causes of vulnerability while increasing resilience across sectors^1^. In low- and middle-income countries (LMICs), disasters frequently worsen already existing socioeconomic inequities, impair health systems, and disrupt livelihoods^3^. The impacts of these disasters are made worse by weak infrastructure, insufficient early warning systems, and insufficient inter-sectoral coordination^4^. As a result, the capacity to undertake thorough multi-hazard risk assessments, which incorporate several hazard categories and their interconnections, is crucial for decreasing disaster-related losses^5^.

Nigeria presents a multifaceted hazard landscape that includes climate, environmental, biological, and human-caused threats. Environmental risks such as flooding, drought, erosion, and windstorms interact with biological risks such as Lassa fever, cholera, and cerebrospinal meningitis (CSM), creating multilayered risks^6^. Niger State, in Nigeria’s North Central region, is particularly vulnerable due to its wide river systems, agricultural economy, and different biological zones^7^. The Niger and Kaduna rivers, as well as other tributaries, increase flood risk, especially during the peak rainy season^8^. Rain-fed agriculture leaves the local economy extremely vulnerable to seasonal variation and major weather disasters^9^. Furthermore, the state’s porous security environment exacerbates displacement, reduces agricultural output, and impedes disaster response efforts^10^. Despite this vulnerability, existing DRR programs in the state have frequently been hazard-specific, missing the comprehensive viewpoint required for holistic preparedness.

A comprehensive multi-hazard risk assessment framework is required for identifying priority risks, mapping their spatial and temporal patterns, and efficiently allocating resources^11^. Traditional risk assessments in Nigeria have frequently concentrated on single hazards, ignoring the cumulative and cascading consequences that result when numerous hazards occur concurrently or sequentially^12^. For example, severe rains may cause floods, facilitating cholera outbreaks while also hindering access to hospitals and markets^13^. The World Health Organization’s Strategic Tool for Assessing Risks (WHO STAR) offers a structured approach for integrating hazard identification, likelihood estimation, impact assessment, and capacity evaluation into a unified procedure. STAR stresses inclusivity, multi-sectoral collaboration, and evidence-based prioritizing, making it well-suited for subnational contexts with varying hazard profiles. It has been used in both high- and low-resource contexts to aid in planning for epidemics, natural disasters, and complex situations^14^.

Despite Niger State’s exposure to a variety of risks, no previously published study has used the STAR methodology to create an integrated risk assessment for the state. Previous studies have focused on epidemiological surveillance for certain diseases^15,16^ or environmental hazard mapping in flood-prone areas^8,17^. These walled approaches impede decision-makers’ ability to plan for concurrent risks or coordinate responses across sectors. Furthermore, the absence of a unified, evidence-based hazard prioritization process impedes resource allocation and undermines resilience-building. By applying the STAR tool, this study seeks to fill that gap, providing a replicable model for other Nigerian states and similar contexts. The assessment engages stakeholders from multiple ministries, departments, and agencies (MDAs) in Niger state, alongside technical partners, thereby fostering inter-sectoral ownership of both the process and its outputs.

The overall aim of this study was to improve disaster risk management in Niger state using the WHO STAR tool, hence increasing preparedness, resilience, and evidence-based decision-making. Specific objectives were to:

1. Conduct a full multi-hazard risk assessment in Niger state, Nigeria, using the WHO STAR tool to identify potential hazards and vulnerabilities.
2. Prioritize hazards based on their likelihood and impact to guide resource allocation and planning.
3. Provide recommendations to support preparedness planning based on prioritized hazards.

## Methods

### Study design

This study employed a cross-sectional design using the World Health Organization (WHO) Strategic Tool for Assessing Risks (STAR) to identify, analyse, and prioritize hazards across biological and non-biological domains. The STAR tool is a standardized framework designed to help identify, analyse, and prioritize multi-hazard risks^14^. The tool uses both quantitative and qualitative methodologies to assess hazards based on their chance of occurrence, possible impact, susceptibility of impacted populations, and institutions’ ability to cope and respond. The assessment was carried out throughout a five-day workshop in Minna, Niger State, from May 13 to 17, 2025, organized by Sydani group in partnership with Nigeria Centre for Disease Control and Prevention (NCDC) with technical support from the Niger state Ministry of Health and the Niger state Emergency Management Agency.

### Description of the WHO STAR and its components

The World Health Organization’s (WHO) Strategic Tool for Assessing Risks (STAR) is a comprehensive risk assessment system that helps identify, analyse, and prioritize potential hazards and risks. The tool offers a systematic and standardized risk assessment approach, allowing for the development of evidence-based risk reduction and management solutions.

The study used the WHO STAR to conduct a complete multi-hazard risk assessment in Niger State, Nigeria. The tool was used to help identify, analyse, and prioritize potential hazards and risks in the state. The WHO STAR consists of several key components including:

4. Hazard Identification: Participants listed potential hazards likely to trigger a state-level response across four domains: natural, biological, technological, and societal. These hazards were identified by stakeholders, literature review, available data, and expert opinions.
5. Risk Analysis: A standardized matrix was used to assess the likelihood and potential impact of identified hazards using historical occurrence, predictive data, and expert judgment.
6. Risk Prioritization: The study team selected detected hazards based on likelihood, potential impact for public health, infrastructure, economy, and environment, to focus on the most critical ones.
7. Capacity Assessment: Policy, institutional, and technical risk management capacities were evaluated to identify gaps.
8. Risk Management Options: Risk management options were identified and assessed, including preventive, preparedness, response, and recovery measures. The acquired data were analysed and interpreted using the WHO STAR tool’s standard framework. The risk assessment results were utilized to help develop suggestions for risk reduction and management measures in Niger State.

**Fig. 1:**
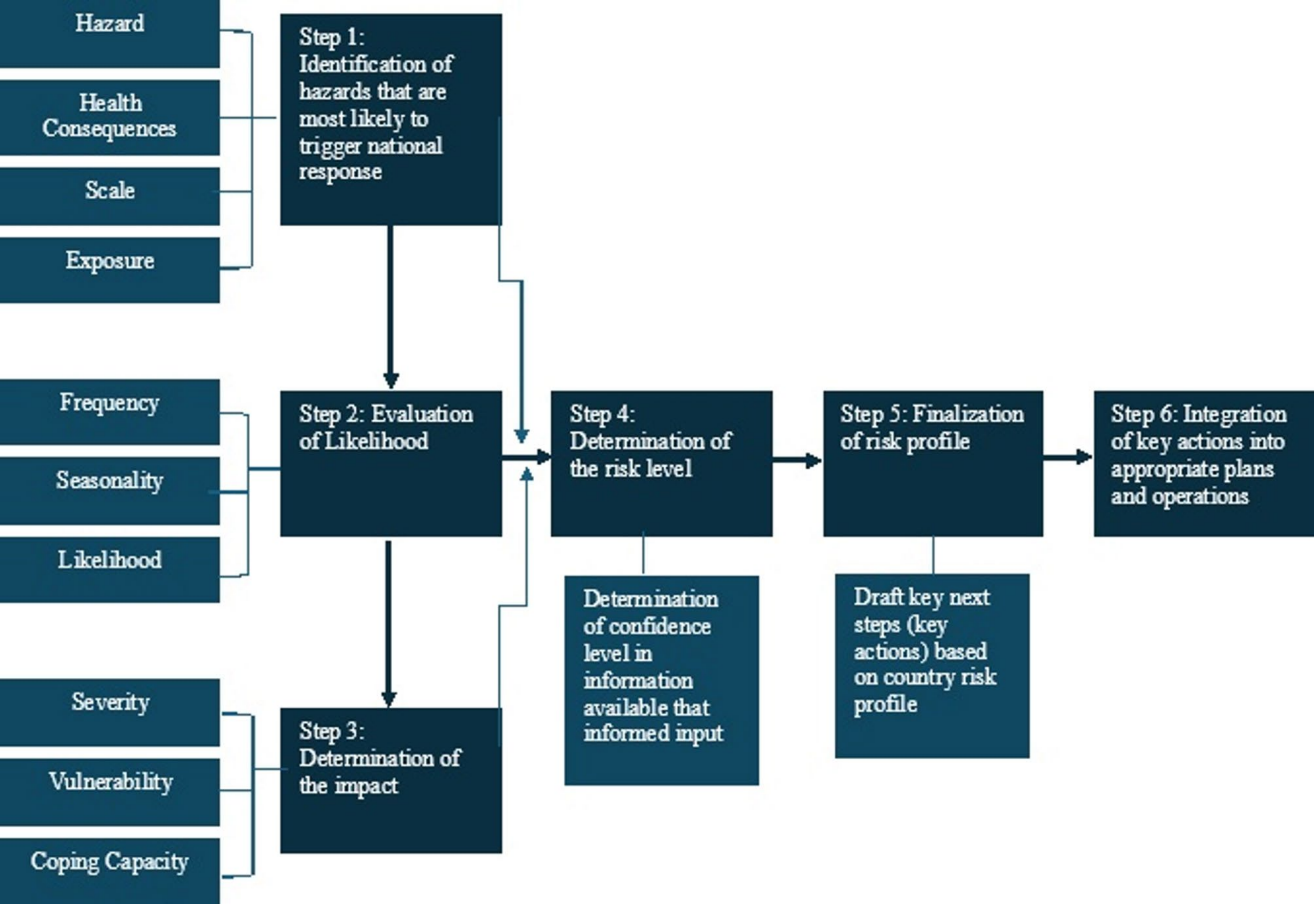
STAR methodology steps.

The detailed methodological workflow is presented in Fig. 1.

### The STAR methodology

The STAR methodology guides countries and subnational levels through a structured process for understanding and prioritizing public health risks. The tool relies on multi sectoral expertise, facilitated discussions, and a standardized scoring criteria. The steps summarize how the methodology works, aligned with the six steps of carrying out a strategic risk assessment.

#### Step one: identification of the hazards

The first step is to identify the hazards that may affect or have affected the country/state. During the workshop, participants validated and refined the list of hazards by drawing on their knowledge of past events, sector-specific information, and data available. This helps ensure that all relevant hazards (natural, biological, technological, and societal) are included before scoring begins.

#### Step two: evaluation of likelihood

Once the hazards have been identified and confirmed, stakeholders worked in small groups to score how likely each of the mentioned hazards occur. The scoring is guided by predefined criteria in the STAR tool, supported by historical data, routine surveillance, and expert judgement. Group discussions help ensure that likelihood scores reflect shared understanding rather than individual opinions (Table 1).

**Table 1:** Overview of likelihood assessment categories in the STAR approach.

Level	Description
Almost certain	It is likely that the hazard will occur in the next 12 months in most circumstances (e.g., probability of 95% or more)
Very likely	It is likely that the hazard will occur in the next 12 months in most circumstances (e.g., a probability of between 70% and 94%)
Likely	The hazard could occur in the next 12 months some of the time (e.g., a probability of between 30% and 69%)
Unlikely	The hazard could occur in the next 12 months some of the time (e.g., a probability of between 5% and 29%)
Very unlikely	The hazard could occur in the next 12 months under exceptional circumstances (e.g., a probability of less than 5%)

#### Step three: determination of the impact

After scoring the likelihood, participants then assessed the potential impact associated with each hazard. This includes considering population exposure, health system capacity, infrastructure, social conditions, and the ability of services to cope if the hazard occurs. Severity, vulnerability, and coping ability are factors which are evaluated independently, and the findings are then used to determine the hazard’s projected impact. The vulnerability criteria in the STAR worksheets structure these discussions, and groups assign scores based on consensus. After the severity, vulnerability and coping capacity scores are determined, the tool automatically calculates the impact score using the formula:

$${\mathrm{Impact}}\;{\mathrm{Score}} = \frac{{{\text{Severity + vulnerability + coping}}\:{\mathrm{capacity}}}}{{\mathrm{3}}}.$$

The impact scoring criteria in the STAR methodology is presented in Table 2.

**Table 2:** Impact scoring criteria in the STAR methodology.

Score	Impact score
1	Negligible
2	Minor
3	Moderate
4	Severe
5	Critical

#### Step four: determination of the risk level

The likelihood and impact scores are entered into the STAR tool (a digital software), which automatically calculates risk levels. This generates a clear risk matrix showing which hazards fall into very high, high, medium, or low categories. The tool also produces visual outputs such as charts and diagrams that help participants interpret the results.

#### Step five: finalization of the risk profile

The scoring outputs are reviewed with participants to confirm accuracy. Any inconsistencies or unclear scores are revisited through discussions. This validation step ensures that the final risk profile truly reflects the collective judgement of the subject matter expert stakeholders. The finalized profile provides a structured picture of priority hazards and the factors driving their risk levels.

#### Step six: integration of key actions into plans and operations

The final step involves using the prioritized hazards to guide preparedness and planning. Participants identify the actions that need to be integrated into emergency plans, sectoral strategies, and routine operations. This helps governments and partners align resources, strengthen systems, and address the risks that pose the greatest threat to the population.

### Study setting

Niger State, located in north-central Nigeria, is the country’s largest state by landmass (76,363 km^2^) and has a population of over six million people scattered throughout 25 Local Government Areas^18^. Its terrain contains significant rivers such as Niger and Kaduna, which makes it prone to flooding, while its agrarian economy is heavily reliant on rainfall, making it subject to droughts and climate variability. Furthermore, recurring insecurity (banditry and kidnapping) and illness outbreaks (cholera, Lassa fever, and meningitis) heighten the state’s risk profile^19^.

The STAR assessment followed a structured timeline with a pre workshop engagement two months before workshop. This involved advocacy visits to key ministries and agencies, initial stakeholder identification, and planning meetings. Identification of eligible institutions, confirmation of representatives, and development of a sector balanced participant list was done one month to the workshop. The workshop was held over a 5-day period from 13th to 17th of May,2025.

### Stakeholder mapping

Stakeholders were purposively selected by the research team using the WHO STAR methodology to ensure broad representations from sectors relevant to disaster risk management^14^. Stakeholders were selected from 38 Ministries, Departments, and Agencies (MDAs) within Niger state, complemented by national level experts from the Nigeria Centre for Disease Control (NCDC), development partners, civil society organizations, and technical experts. Selection criteria included institutional mandates and operational experience in disaster risk management, health, environment, or security; at least three years of operational or technical experience; direct involvement in emergency response, surveillance or risk management, and ability to provide sector specific perspectives during the assessment. In total the workshop had over 50 participants which included experts from the following organizations:

- Public health: Niger State Ministry of Health, NCDC, WHO.
- Disaster management: Niger State Emergency Management Agency (NSEMA), Federal Road Safety Corps (FRSC), National Emergency Management Agency (NEMA).
- Agriculture and environment: State Ministry of Agriculture, Forestry Commission.
- Security and law enforcement: Police, Civil Defence Corps.
- Infrastructure and planning: Ministry of Works, Urban Development Authorities.
- Civil society organizations and community-based groups.

See Supplementary Material 1 for details. This multidisciplinary composition ensured coverage of all major hazard domains.

## Workshop preparation and training of participants

Before the workshop, pre-workshop advocacy visits and planning meetings were held with the Niger State Ministry of Secondary and Tertiary Health, Niger State Ministry of Primary Health Care, Ministry of Environment, the Nigeria Centre for Disease Control and Prevention (NCDC), and partners such as WHO and UNICEF. These discussions ensured political support, established goals, and aligned expectations.

Before data collection, participants got training on the STAR approach. The training lasted half a day and included presentations on hazard typologies, the STAR assessment process, and rating criteria for likelihood, impact, susceptibility, and coping capacity. Practical activities and moderated group discussions were utilized to increase familiarity with the tool and ensure uniform application of scoring standards. The training was facilitated by technical officers from Sydani Group and the NCDC who had previous experience applying STAR at other subnationals. The training ensured that participants had a shared understanding of the assessment framework and scoring expectations.

### Data collection procedures

The assessment combined primary and secondary data sources.

#### Primary data source

A structured plenary and breakout group discussion was held with stakeholders to identify and characterize hazards. Participants were grouped into three groups, depending on the sectoral mandates (such as health, environment, and security). Technical facilitators from Sydani group and NCDC provided expert guidance on hazard classification and scoring.

During hazard identification, participants listed hazards likely to trigger a state level response based on past experiences and available evidence. Facilitators guided discussions to ensure clarity and confirm alignment with STAR definitions. The groups rated the likelihood of each based on historical occurrence, frequency patterns, and available surveillance or disaster records. Next, they scored impact severity using STAR criteria that consider consequences for health, essential services, infrastructure, and economy. After likelihood and impact scoring, participants assessed vulnerability which reflects the degree to which populations or systems can be harmed. Finally, coping capacity was scored by evaluating existing preparedness measures, emergency response structures, and institutional capabilities.

All scoring activities were moderated to ensure consistency and adherence to STAR guidelines. After group scoring, the facilitators used iterative review and voting to create consensus on hazards rankings and criteria. Results were reviewed in plenary to reach an agreement. Discrepancies were resolved through further discussion and reference to documented evidence.

#### Secondary data source

Secondary data provided context and supported evidence for scoring. A review of relevant studies, academic literature, and official data on risks in Niger state was conducted by a team of public health analysts, epidemiologists, and environmental specialists who were part of the workshop participants. The review covered the period from 2020 to 2024 and included national surveillance data, state disaster records, meteorological reports, and peer reviewed publications. Analysing this historical disaster data from state emergency records, disease surveillance systems, and meteorological agencies helped determine hazard frequency and seasonality. These secondary data were obtained from the following sources;

- Historical disaster records from NSEMA, NEMA, and the Niger state ministry of health.
- Surveillance data from the Nigeria Centre for Disease Control (NCDC).
- Meteorological data from the Nigerian Meteorological Agency (NiMET).
- Published literature on disaster risk and resilience in Nigeria.
- UN, WHO, and IFRC reports on hazards and emergencies in Nigeria and West Africa.

The review team searched online databases such as PubMed, Google Scholar using keywords related to flooding, cholera, drought, insecurity, and multi-hazard risk.

### Hazard identification

Participants brainstormed and reviewed documents to create an initial list of hazards relevant to Niger State. The hazards were grouped into four STAR domains:

- Natural hazards (e.g., floods, drought, rainstorms, lightning).
- Biological hazards (e.g., cholera, Lassa fever, meningitis, measles, Acute flaccid paralysis).
- Technological hazards (e.g., industrial accidents, road traffic crashes, boat mishaps).
- Societal/security hazards (e.g., armed banditry, kidnapping, communal conflict).

A total of 18 hazards were identified for further analysis.

Each hazard was assessed along four dimensions;

9. Likelihood of occurrence: Probability that the hazard will occur in the future, based on historical trends, surveillance data, and expert opinion. This was scored on a 5-point scale (1 = very unlikely, 5 = very likely).
10. Impact severity: Potential consequences on health, livelihoods, infrastructure, and governance. This was scored on a 5-point scale (1 = negligible, 5 = catastrophic).
11. Vulnerability: Degree to which populations, systems, and sectors are susceptible to harm, considering socio-economic conditions, environmental exposure, and resilience factors. This was scored qualitatively and ranked.
12. Coping Capacity: The ability of state institutions, communities, and systems to prevent, prepare for, and respond to the hazard. This was scored qualitatively and ranked.

Scores for likelihood and impact were multiplied to generate a risk index for each hazard. Hazards were then categorized into four priority categories: *very high risk*,* high risk*,* moderate risk*,* and low risk.*

After individual scoring, facilitated plenary discussions were held to reach an agreement. Hazards were put on a likelihood-impact matrix to help visualize priority hazards. Vulnerability and coping capacity were then used to contextualize the rankings. Hazards classified as very high risk were those with both high probability and severe potential consequences, compounded by high susceptibility and inadequate coping capacity.

To enhance operational relevance, hazards were further analysed for:

Seasonality: Participants mapped hazard occurrence against the calendar year, producing a seasonal hazard calendar (e.g., flooding in July–September, cholera peaks during the rainy season, meningitis in the dry season).

Geographic Distribution: Hazards were mapped by Local Government Areas (LGAs), highlighting hotspots such as riverine LGAs (flooding, boat mishaps) and northern LGAs (banditry).

The distribution and analysis of hazards in the evaluation were based on data at the Local Government Area level, while the overall prioritizing was based on the risk profile at the state level.

Draft findings were presented to stakeholders for validation, and discrepancies were resolved through consensus, ensuring that the final prioritization reflected both evidence and collective expertise.

### Validation of results

At the end of the workshop, preliminary hazard rankings were presented to all participants for review. Stakeholders confirmed that the results aligned with available evidence and field realities, this served as a form of participant checking and strengthened the validity of the assessment.

### Data analysis

Microsoft Excel was used for data management and calculation of risk scores; likelihood and impact scores were multiplied to create composite risk scores. Descriptive statistics summarized hazard distribution, number of affected Local Government Areas, and seasonal patterns. Seasonal calendars and risk matrices were generated using STAR templates. In addition, qualitative notes from discussions were thematically analyzed to provide context for hazard classification, vulnerability patterns, and coping capacity. Results were synthesized to produce an integrated risk profile for Niger state.

### Quality assurance

Quality assurance measures included cross verification of data entries, facilitator oversight during scoring sessions, alignment with STAR guidelines^14^, and assignment of confidence ratings for each hazard. To improve reliability, each hazard scoring exercise was conducted in facilitated groups and validated during plenary sessions. Facilitators cross-checked data entries against documented evidence, and discrepancies were resolved through consensus. Confidence levels for each hazard score were assigned (good, satisfactory, unsatisfactory) based on data availability and quality.

## Results

### Overview of identified hazards

The multi-hazard risk assessment conducted in Niger State identified 18 hazards across biological, environmental, technological, and security. These hazards were selected following multi sectoral consultations, review of surveillance and disaster records, and group consensus during the STAR workshop. These hazards were carefully categorized using the WHO STAR technique based on their chance of occurrence, impact, potential, and coping capacity. The study presented a holistic view of hazards affecting the state and their distribution across different local government areas (LGAs).

Available surveillance, meteorological, and disaster management reports provided additional context for several of the priority hazards identified. Flooding has consistently been one of the most widespread hazard in Niger state, affecting an estimated 15 to 19 LGAs annually over the last five years, particularly those along the Niger and Kaduna river. Cholera outbreaks have been recorded in at least 8 to 12 LGAs each year, with seasonal peaks during the rainy season when contamination of water is most pronounced. Security-related hazards, especially banditry and kidnapping continue to drive significant population displacement, with several thousand persons affected annually, mainly in the northern LGAs that share borders with Kaduna, Zamfara, and Kebbi states. These descriptive patterns align with stakeholders assessments during the STAR workshop and helped inform the final prioritization of hazards.

### Risk classification of hazards

**Fig. 2:**
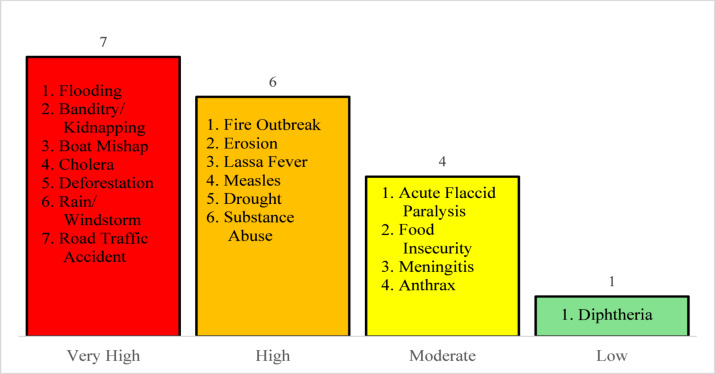
Risk level of hazard in Niger state, 2025.

Using the STAR thresholds, hazards were classified into four risk categories. Seven hazards were classified as very high risk, six as high risk, four as moderate risk, and one as low risk. Flooding, banditry/kidnapping, boat disasters, cholera/acute watery diarrhoea, road traffic accidents, deforestation, and rain/windstorms were all considered very high risk. High-risk hazards included fires, Lassa fever, measles, drought, substance abuse, and erosion. Acute flaccid paralysis, meningitis, food insecurity, and anthrax were all classified as moderate risks, while diphtheria was considered a low risk. Figure 2 shows the risk levels of the hazards identified. These hazards had a combination of high likelihood, severe impact, and limited coping capacity.

### Geographic distribution of hazards

The assessment also revealed significant regional groupings, which frequently aligned with natural factors, socioeconomic patterns, and security dynamics. Flooding affected 19 LGAs, primarily along the Niger and Kaduna rivers, including Agaie, Bida, Lapai, Lavun, and Mokwa. During the rainy season, these areas experience recurring flooding, affecting agriculture and settlements. Banditry/kidnapping was concentrated in nine LGAs (Borgu, Mariga, Mashegu, Rafi, Shiroro, Wushishi, Kontagora, Muya, and Paikoro), mostly in the north and northwest due to forested terrain and limited law enforcement presence. Boat mishaps were concentrated in six LGAs (Agwara, Borgu, Katcha, Mokwa, Shiroro, and Wushishi). Cholera outbreaks occurred in both urban and rural LGAs, indicating insufficient WASH infrastructure, while Gurara, Kontagora, Magama, Mokwa, Shiroro, and Wushishi experienced the most severe drought due to rain-fed agriculture and little irrigation infrastructure (Table 3).

**Table 3:** Geographical areas affected by hazards in Niger state, 2025.

Hazard category	Hazard	Risk level	Affected areas (LGAs)
Biological	Cholera/Acute watery diarrhea	Very high	Agaie, Agwara, Bida, Bosso, Chanchaga, Edati, Gurara, Katcha, Kontagora, Lapai, Lavun, Magama, Mariga, Mashegu, Mokwa, Muya, Paikoro, Rafi, Rijau, Shiroro, Suleja, Tafa, Wushishi
	Lassa fever	High	Bida, Suleja, Tafa
	Measles	High	Agaie, Agwara, Bida, Bosso, Chanchaga, Edati, Gbako, Gurara, Katcha, Kontagora, Lapai, Lavun, Magama, Mariga, Mashegu, Mokwa, Muya, Paikoro, Rafi, Rijau, Shiroro, Suleja, Tafa, Wushishi
	Meningitis	Moderate	Bida, Bosso, Chanchaga, Edati, Gbako, Gurara, Katcha, Kontagora, Lavun, Magama, Mokwa, Paikoro, Rijau, Suleja, Tafa
	Diphtheria	Low	Bida, Kontagora, Suleja, Tafa, Mariga
	Acute flaccid paralysis	Moderate	Agaie, Agwara, Bida, Bosso, Chanchaga, Edati, Gbako, Gurara, Katcha, Kontagora, Lapai, Lavun, Magama, Mariga, Mashegu, Mokwa, Muya, Paikoro, Rafi, Rijau, Shiroro, Suleja, Tafa, Wushishi
Environmental	Flooding	Very high	Agaie, Bida, Borgu, Bosso, Chanchaga, Edati, Gbako, Katcha, Kontagora, Lapai, Lavun, Mariga, Mashegu, Mokwa, Rafi, Rijau, Shiroro, Suleja, Wushishi
	Drought	High	Gurara, Mokwa, Shiroro, Kontagora, Magama, Wushishi
	Erosion	High	Agaie, Bosso, Chanchaga, Katcha, Kontagora, Lapai, Mashegu, Mokwa, Tafa
	Rain/Windstorm	Very high	Agaie, Agwara, Bosso, Chanchaga, Gbako, Kontagora, Lapai, Lavun, Mariga, Mashegu, Mokwa, Paikoro, Rijau, Shiroro, Suleja, Wushishi
Security/ Societal	Banditry/Kidnapping	Very high	Borgu, Kontagora, Mariga, Mashegu, Muya, Paikoro, Rafi, Shiroro, Wushishi
	Substance abuse	High	Chanchaga, Kontagora, Suleja, Mariga
Technological	Road traffic accidents	Very high	Agaie, Bida, Bosso, Chanchaga, Gurara, Kontagora, Lapai, Mokwa, Suleja
	Fire outbreaks	High	Agaie, Bida, Borgu, Bosso, Chanchaga, Kontagora, Lapai, Mokwa, Rafi, Shiroro, Suleja
	Boat mishaps	Very high	Agwara, Borgu, Katcha, Mokwa, Shiroro, Wushishi
Others	Deforestation	Very high	Edati, Lapai, Lavun, Mokwa, Wushishi
	Food insecurity	Moderate	Agaie, Agwara, Bosso, Lavun, Magama, Rafi, Shiroro
	Anthrax	Moderate	Suleja

### Seasonal patterns of hazards

**Fig. 3:**
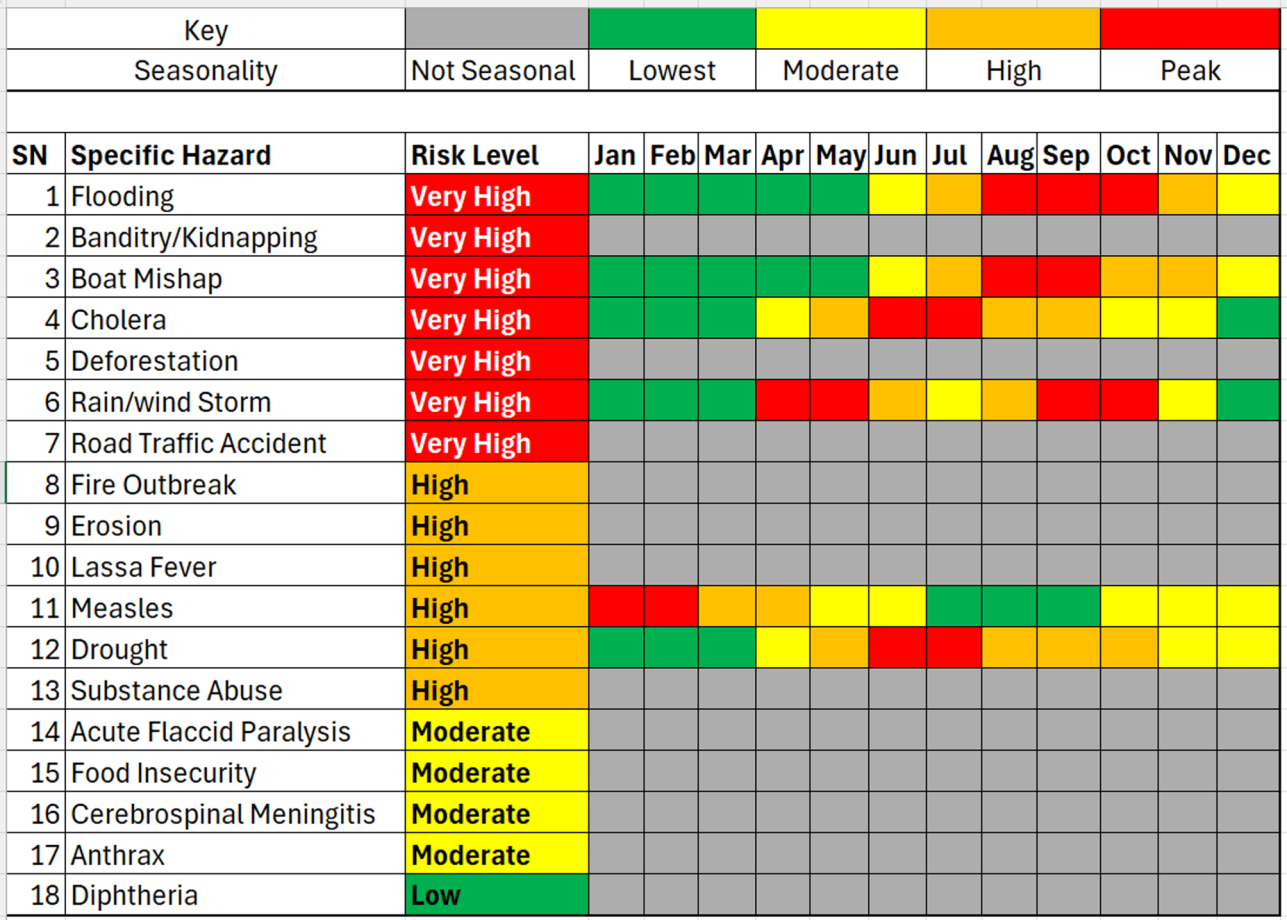
Niger state hazards risk calendar, 2025 (generated by WHO STAR).

Seasonality analysis represented in Fig. 3 revealed that certain hazards show predictable patterns; Flooding occurs between July and October, with start in June, and this is as a result of the high rainfall occurring in these months. Peak flooding months have the highest number of boat mishaps due to increased river traffic and hazardous navigation conditions. Cholera epidemics typically occur during the rainy season, peaking between June and September, and are generally linked to flooding caused by contaminated water, while droughts peak between June and November in years with delayed or unpredictable rainfall, affecting crop production and livestock productivity. For Rain/windstorms, they are more common in transitional months (May-June, September-October). Biological hazards such as measles cases occur during the dry season (December-March), when mobility increases and vaccine coverage gaps become more visible. These seasonal variations were consistently highlighted during stakeholder discussions and aligned with historical surveillance and meteorological data. The seasonality of these hazards highlights opportunities for anticipatory action, such as pre-positioning supplies, scaling up public health messaging, and reinforcing early warning systems.

### Likelihood and impact scoring

**Fig. 4:**
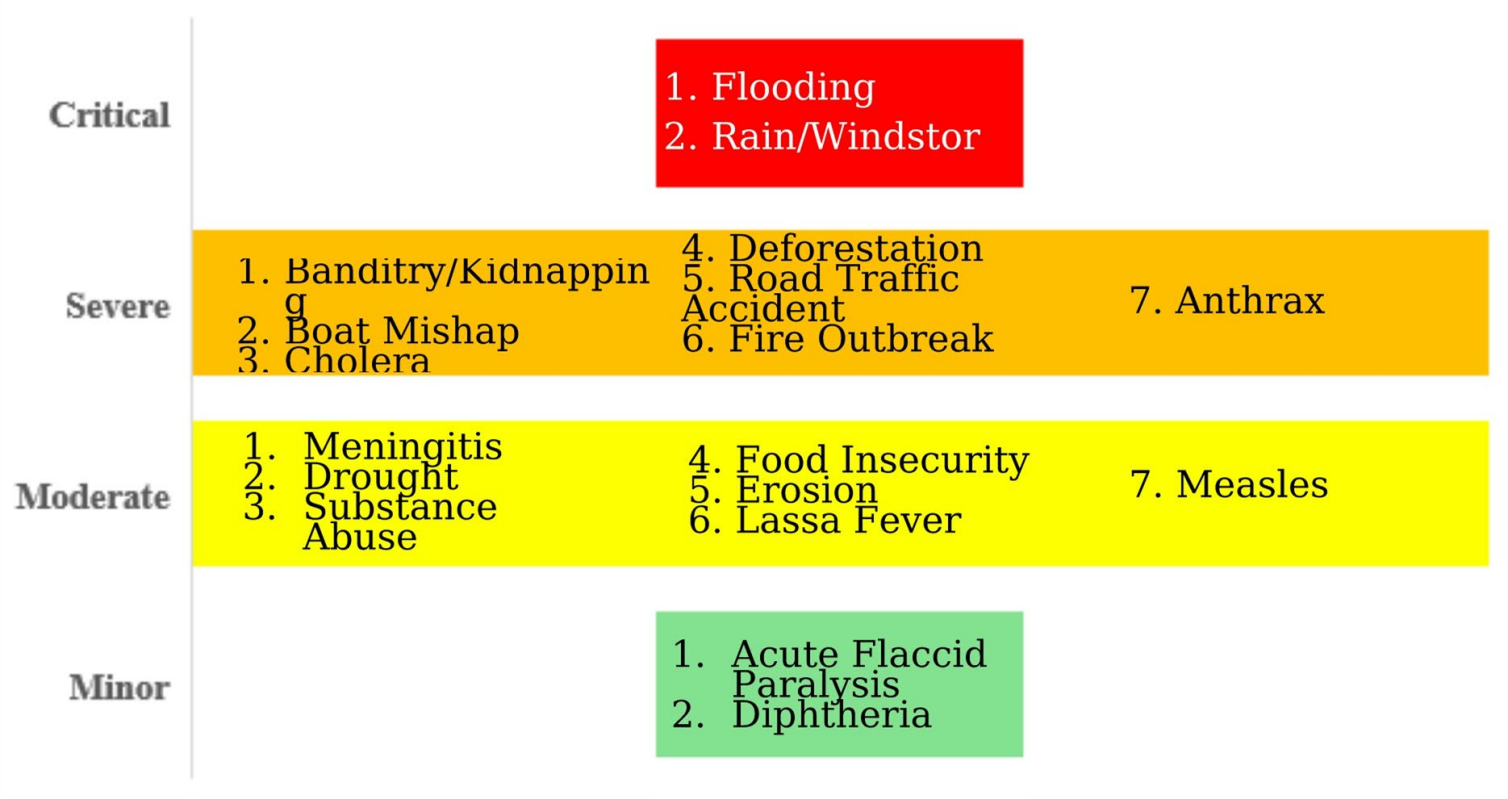
Impact of hazards in Niger state. 2025.

**Fig. 5:**
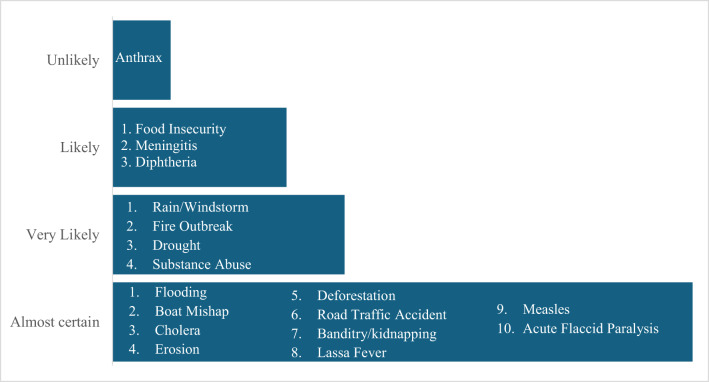
Likelihood of occurrence of hazards in Niger state. 2025.

**Fig. 6:**
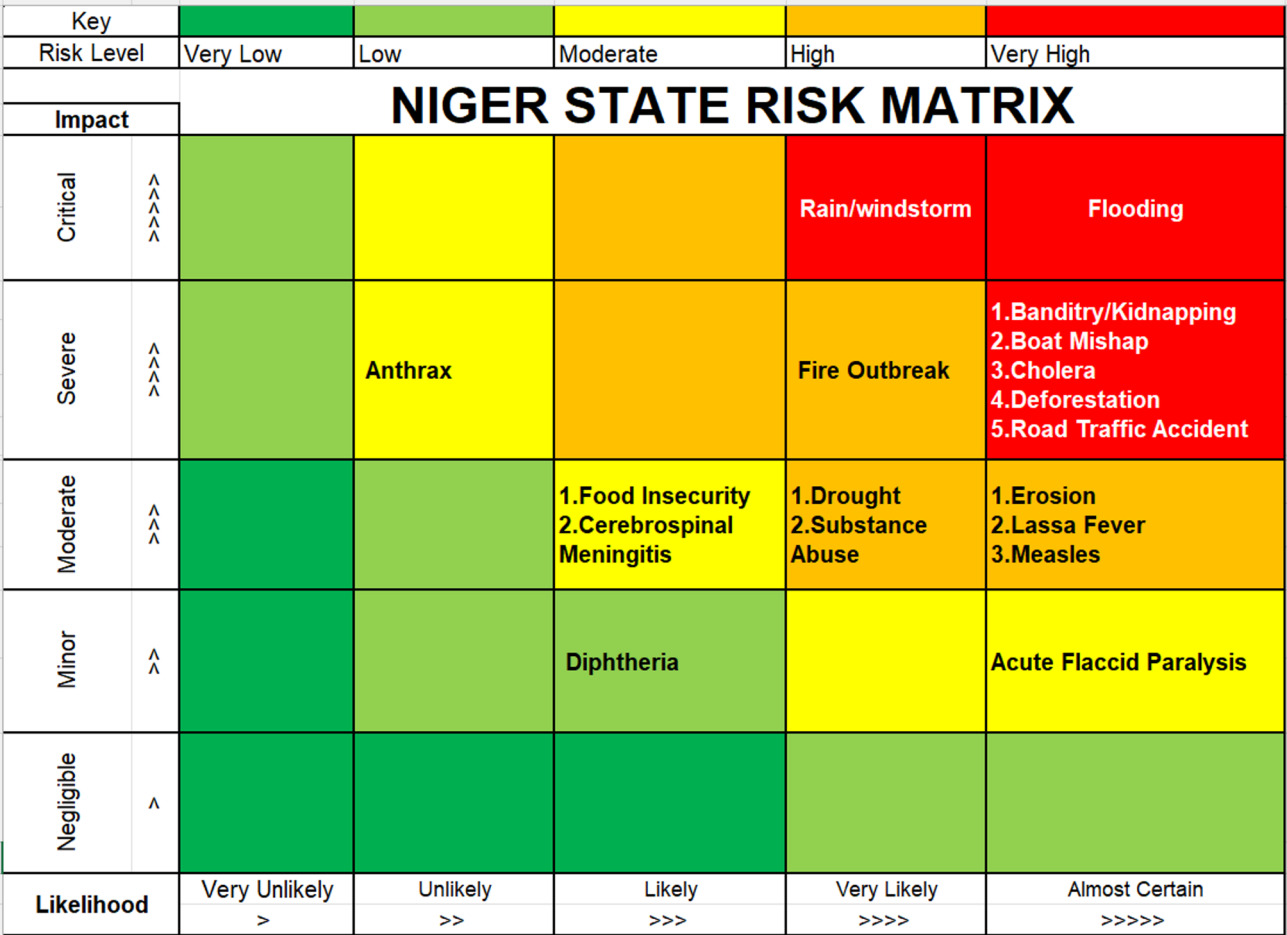
Risk matrix of hazards in Niger state. 2025 (generated by WHO STAR).

Impact and likelihood scores (Figs. 4 and 5) varied across hazards. Flooding, banditry/kidnapping, cholera, and boat accidents had the highest likelihood scores, reflecting their frequent recurrence in the state. Flooding had significant repercussions, including loss of life, population displacement, and infrastructure destruction. Impact scores were highest for security threats, cholera, and boat mishaps due to their documented consequences on health, infrastructure, displacement, and essential services. These patterns are illustrated in the STAR likelihood and impact matrix (Fig. 6). Environmental hazards such as deforestation and erosion have long-term repercussions, leading to vulnerability rather than urgent emergencies.

### Vulnerability and coping capacity

Vulnerability Patterns showed that rural and riverine areas were more vulnerable due to limited access to functional health services, poor road and transportation infrastructure, which causes delays in emergency response, strong reliance on climate-sensitive livelihoods, low literacy, and risk knowledge in some communities, which hinders preparedness efforts. Social vulnerability was worsened in conflict-affected LGAs, where insecurity restricted humanitarian access and displaced communities from arable land.

**Fig. 7:**
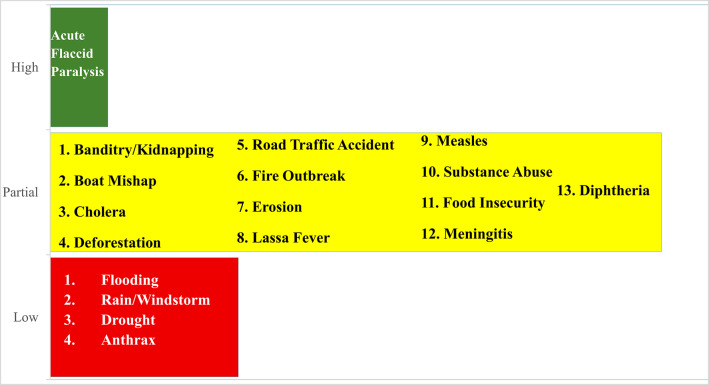
Coping capacity of Niger state to hazards. 2025.

Coping capacity was also assessed to understand how communities cope with some of these hazards (Fig. 7), it was rated low or partial for eleven of the eighteen hazards. Coping ability was limited for flooding, drought, and rain/windstorm due to under-resourced WASH services, inadequate drainage infrastructure, and gaps in emergency services. Fire outbreaks, erosion and others have low reaction capability due to limited scale and coordination. However, established polio surveillance systems and vaccine efforts resulted in high capacity for acute flaccid paralysis. In most cases, institutional readiness was hindered by fragmented planning, inadequate inter-agency cooperation, and reliance on external donor support for response activities. High coping capacity means that although all coping mechanisms necessary for the hazard are present, they have never been evaluated in a simulated exercise or under real-world stress conditions, a moderate/partial coping capacity means there are some coping mechanisms that are necessary for the hazard, but their functioning and sustainability have not been guaranteed, for example, by being incorporated into the national health sector plan’s operating plan with a reliable source of finance, and finally a low coping capacity means that human, material, strategic, and financial core coping capacities needed for the hazard are still at the developmental stage.

Certain attributes have been attained and others have begun to be implemented.

### Confidence ratings

**Fig. 8:**
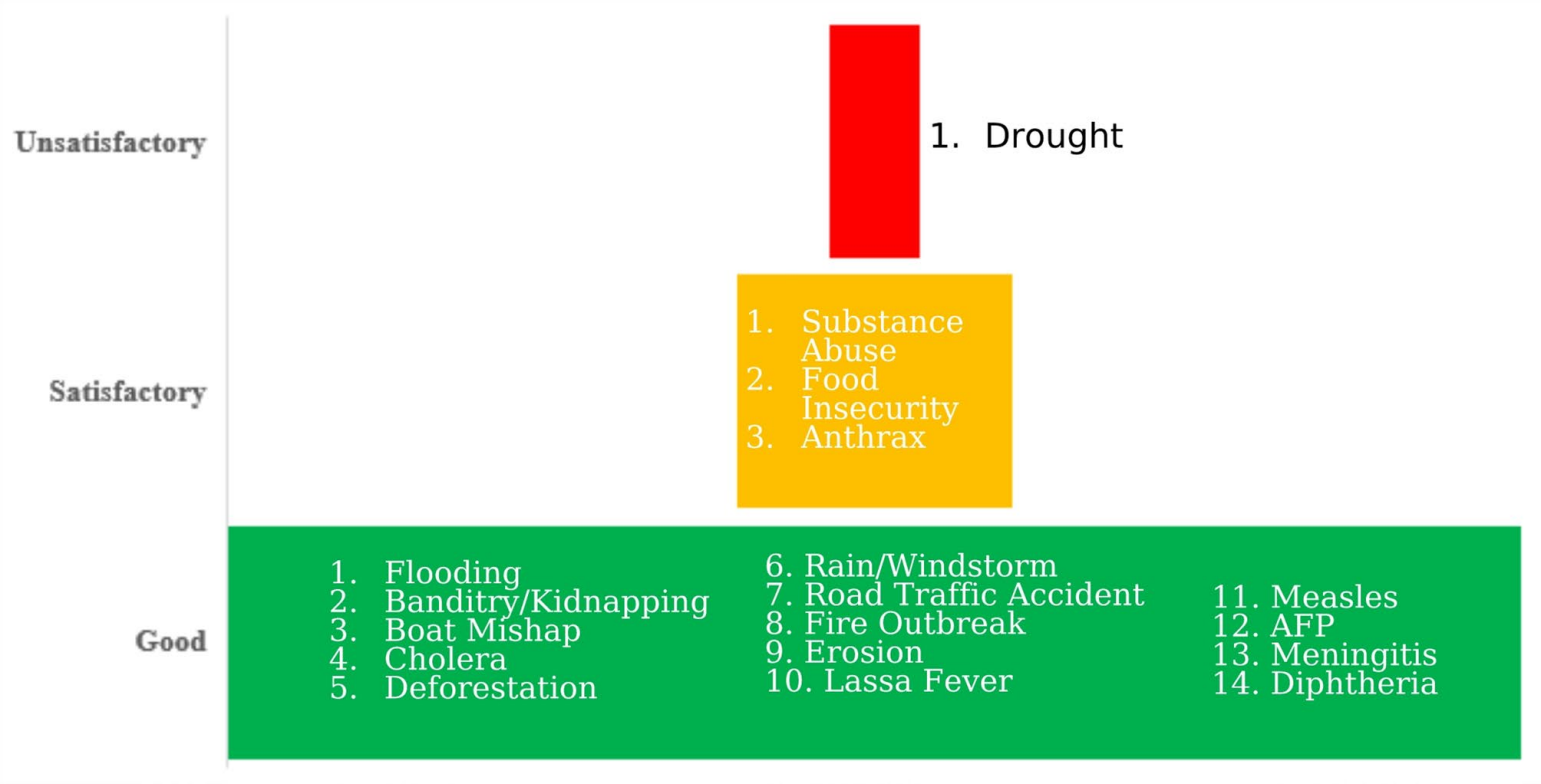
Confidence level of data source.

Confidence ratings for each hazards assessment ranged from good to unsatisfactory (Fig. 8). Data confidence ratings were generally high for hazards with established surveillance and reporting systems (e.g., cholera, AFP, and measles). However, hazards with limited documentation were rated as satisfactory, and drought confidence was low, owing to irregular meteorological records and inadequate integration of agricultural produce data into risk monitoring. These ratings are presented in Fig. 8.

### Summary of priority hazards

The combined analysis identified seven very high-risk hazards represented in Supplementary Material 2 requiring urgent and ongoing preparedness efforts. These hazards were consistently supported by historical data, expert judgement, and geographical and seasonal patterns. Combining likelihood, impact, susceptibility, and coping capacity ratings, the following top five risks emerged as priority risks in Niger state: Flooding, banditry/kidnapping, boat mishaps, cholera/acute watery diarrhea, and rain/windstorms. These hazards not only had the highest overall risk, but they also showed cascading effects, with the ability to cause additional hazards and worsen disasters.

Finally, some cross-cutting findings were evident, for example, flooding precipitated cholera outbreaks, drought leading to food insecurity, and insecurity causing disease outbreak responses. Seasonal regularity of hazards presents opportunities for early warning and proactive preparedness. It was also noticed that community-level resilience remains underdeveloped, with most preparedness actions occurring at the institutional level rather than at the household level, and limited integration of health, environmental, and security data into a single decision-making framework, resulting in sectoral silos.

## Discussion

This study employed the WHO Strategic Tool for Assessing Risks (STAR) to conduct the first comprehensive, state-level multi-hazard risk assessment in Nigeria. A total of 18 hazards were identified, with seven categorized as extremely high risk and six as high risk. Flooding, cholera, banditry/kidnapping, road traffic accidents, and boat accidents ranked as the most dangerous hazards to public health and safety. Several risks, including flooding, cholera, boat accidents, and drought, exhibited strong seasonal patterns related to rainfall and river dynamics. The geographic distribution showed two LGAs of concern, riverine LGAs, which were particularly prone to flooding and boat accidents, and northern LGAs, where insecurity from banditry and kidnapping was concentrated.

Rural, riverine, and conflict-affected LGAs were the most vulnerable, with inadequate coping capacity for environmental and security hazards. These findings contribute to the study’s goal of developing an actionable, evidence-based risk profile that can guide Niger State’s readiness, mitigation, and response efforts.

The prevalence of floods in Niger State is consistent with patterns recorded in other studies. Previous studies^20–22^ have consistently identified floods as an annual hazard with serious repercussions for agriculture, livelihoods, and infrastructure. Floods frequently result in secondary health crises, most notably cholera outbreaks, which have been observed in various communities in Niger state^15,23^. Our data support this link while also placing cholera in a larger multi-hazard framework, emphasizing the importance of coordinated WASH and flood management methods. The high ranking of insecurity, particularly banditry and kidnapping, reflects national trends. According to reports from the United Nations Development Programme (UNDP, 2023) and the Office for the Coordination of Humanitarian Affairs (OCHA, 2023), rural violence is growing in north-central Nigeria, undermining disaster preparedness and limiting humanitarian access^24,25^. Unlike most previous DRR evaluations, our study formally incorporated insecurity into hazard prioritization, advancing the discipline by illustrating how war and disaster risk are deeply intertwined. Seasonal trends for cholera, boat mishaps, and drought are comparable with epidemiological and meteorological literature from Nigeria and West Africa^26,27^. Linking these seasonal peaks to individual LGAs gives operationally relevant data for early warning and preparedness. In addition, the discovery of insufficient coping capacity, particularly for drought and flooding, is consistent with findings from the United Nations Office for Disaster Risk Reduction (2019), which highlight inadequate contingency planning, poor inter-agency coordination, and underfunded infrastructure in resource-limited settings^28^.

The high-risk hazards found in Niger State are caused by a mix of environmental, social, and structural factors. The state’s geology, characterized by rivers and floodplains, makes flooding unavoidable after heavy rains, and climate change has increased rainfall variability, resulting in more frequent and severe floods^29^. Urban flooding is worsened by inadequate drainage and waste management, and cholera is fuelled by inadequate WASH facilities, unclean water, and overcrowded living situations, especially in peri-urban areas^30,31^. Insecurity stems from larger national and regional crises such as porous borders, inadequate law enforcement, the proliferation of firearms, and pervasive poverty, all of which contribute to youth engagement in crime. This has caused displacement, reduced access to healthcare, and disrupted agriculture^32^. Poor infrastructure, ineffective traffic enforcement, and a lack of trauma treatment capacity all contribute to road traffic accidents. These drivers demonstrate how interrelated vulnerabilities form an ecosystem of overlapping risks that overwhelm coping capacity.

Even though this assessment was carried out in a subnational level in Nigeria, the findings are applicable outside the country and add to the larger international conversations on multi-hazard risk assessment^11,33^. Flooding, drought, cholera, and security-related displacement are among the key hazards found in Niger state that are also acknowledged as serious risks in other low- and middle-income countries in Africa, Asia, and Latin America^13,26,34–36^. These risks are a reflection of global trends brought about by socio-economic weaknesses, population expansion, climate change, and weak health systems. Additionally, other countries looking to adapt global risk assessment techniques can gain important insights from the subnational application of the WHO STAR methodology in Nigeria. Many nations face comparable challenges related to limited data availability, reliance on expert consensus, and multisectoral coordination, making the lessons from this study relevant to similar contexts worldwide^37^. By documenting how STAR was implemented in a low-resource setting and demonstrating its potential to inform preparedness planning, this study adds to emerging international evidence on practical approaches for evaluating and prioritizing public health risks in vulnerable regions.

These findings have significant implications for policy, health systems, and disaster preparedness. First, STAR-based prioritizing enables Niger State to shift from reactive crisis management to evidence-based resource allocation, with a focus on the hazards most likely to cause severe harm. Second, the obvious seasonal patterns allow for proactive action, such as strengthening flood defences before the rainy season, increasing WASH interventions ahead of the cholera season, and maintaining boat safety precautions during peak travel months. Third, by considering insecurity in hazard prioritization, this study illustrates the importance of integrating disaster risk reduction and security planning, encouraging collaboration among health agencies, emergency management, and security forces. Fourth, the vulnerability of rural and riverine populations need locally tailored, context-specific interventions: flood-prone LGAs may require early warning systems and boat safety programs, whereas drought-prone LGAs may benefit from climate-smart agriculture and water storage systems. Finally, the assessment’s participatory, multi-sectoral nature demonstrates that collaborative planning is viable and might be institutionalized as a permanent state-level disaster risk reduction platform.

## Strengths and limitations

A major strength of this study is its use of a standardized global tool, the WHO STAR, at the subnational level, exhibiting methodological rigor while adjusting to local realities. The participation of over 50 stakeholders from 38 departments and organizations meant that the findings were founded on diverse expertise, fostering consensus and local ownership. The process also revealed seasonal hazard calendars and regional risk mapping, which are advances that improve operational preparation. Furthermore, by combining health, security, and environmental concerns into a single framework, the study produced a comprehensive perspective rarely seen in Nigerian disaster risk reduction literature.

Despite its strengths, the study has certain limitations that should be considered when interpreting the findings. First, the use of a cross-sectional design means that the analysis reflects hazard patterns at a single point in time, therefore, it only offers a snapshot of hazard patterns. Hazards may fluctuate as a result of climate change, insecurity, or population changes. Data gaps further reduce precision for threats, including drought, substance addiction, food shortages, emerging hazards, and evolving security dynamics may not be fully captured. Second, the scoring process was mainly based on stakeholder perspectives, which, while systematic, introduces subjectivity, even though confidence levels were recorded to limit this, but bias is still possible. Hazards that are most familiar, better documented, or frequently encountered may receive higher attention than slow onset or chronic hazards with limited visibility. Third, the assessment did not include a formal statistical test of scoring consistency. Although extensive facilitation and consensus building were used to strengthen objectivity, quantitative consistency metrics were not calculated. Finally, while the findings are particularly relevant to Niger State, they may not be generalizable without modification to other Nigerian states.

## Directions for future research

Future research should conduct a longitudinal multi-hazard monitoring to detect trends and shifts in hazard profiles over time and expand similar STAR-based assessments to other Nigerian states to allow comparative risk profiling and resource allocation at the national level.

## Conclusion

In conclusion, this study highlighted flooding, cholera, banditry, road traffic accidents, and boat mishaps as Niger State’s greatest hazards to public health and safety. It is the first time the WHO STAR technique has been applied at the subnational level in Nigeria, demonstrating the feasibility of risk-informed state planning. The implications are immediate and clear: improve early warning systems, combine health and security measures, invest in WASH and resilient infrastructure, and prepare clinical services for seasonal surges. Policymakers should incorporate STAR outcomes into Niger State’s emergency preparedness and response strategies, as well as integrate them with national frameworks and the Sendai Framework for Disaster Risk Reduction. Furthermore, repeating the study every 3–5 years will aid in tracking emerging hazards, while expanding the approach to other states can support a national hazard prioritization plan. This will improve Nigeria’s health security, increase resilience to climate and conflict risks, and ultimately save lives and livelihoods.

## Recommendations

Based on the findings of our study, we propose the following recommendations for Niger state MDAs:

Niger State Ministry of Health.

1. Strengthen early warning systems and rapid response teams for cholera, Lassa fever, and other epidemic-prone diseases.
2. Improve WASH services to reduce waterborne disease outbreaks.
3. Expand routine and supplemental immunization campaigns for measles, meningitis, and diphtheria.
4. Improve emergency medical services and trauma care facilities for road traffic accidents and boat mishaps.

Ministry of Environment.

1. Construct and rehabilitate drainage systems in flood-prone communities.
2. Promote climate-resilient agriculture and drought mitigation strategies.
3. Establish and enforce environmental protection measures to reduce deforestation and erosion.

Ministry of Agriculture.

1. Scale up community-level food security programs to reduce the impact of drought and floods on livelihoods.
2. Strengthen animal health surveillance to monitor and prevent zoonotic diseases such as anthrax.

Ministry of Education.

1. Integrate disaster risk reduction education into school curricula.
2. Build community capacity for first response and self-protection in flood and conflict-prone areas.
3. Conduct community sensitization campaigns on safe water use, hygiene, and emergency preparedness.

## Supplementary Information

Below is the link to the electronic supplementary material.

Supplementary Material 1

Supplementary Material 2

## Data Availability

Data used will be available through the corresponding author upon reasonable request.

## Abbreviations

AFENET: African field epidemiology network
AFP: Acute flaccid paralysis
CSM: Cerebrospinal meningitis
DRR: Disaster risk reduction
FRSC: Federal road safety corps
IFRC: International federation of red cross and red crescent societies
LGAs: Local government areas
LMICs: Low and middle income countries
MDAs: Ministries, departments, and agencies
NCDC: Nigeria centre for disease control
NEMA: National emergency management agency in Nigeria
NiMET: Nigerian meteorological agency
NSEMA: Niger state emergency management agency
OCHA: Office for the coordination of humanitarian affairs
STAR: Strategic tool for assessing risk
UNDP: United Nations development programme
UNICEF: United Nations children’s fund
UN: United Nations
WASH: Water, sanitation, and hygiene
WHO: World Health Organization
