# Supplementary material for: Strengthening disaster preparedness and health security in Niger state, Nigeria through a WHO STAR–based multi-hazard risk assessment
Source: Sci Rep. 2026 Jan 16;16:4607. doi: 10.1038/s41598-025-34702-z (PMC12868816; doi:10.1038/s41598-025-34702-z)
Supplement: Supplementary file 2 — Supplementary Material 2 [file 41598_2025_34702_MOESM2_ESM.docx]

*Table 4: Niger State All-hazard Report (generated by WHO STAR)*

| S/n | Title | Risk  Level | Geographical Areas Affected | Estimated  Seasonality (next  3 months) | Estimated  Seasonality (next  4-6 months) | Likelihood | Impact | Vulnerability | Coping  Capacity | Confidence  Level | Date  Updated |
| --- | --- | --- | --- | --- | --- | --- | --- | --- | --- | --- | --- |
| 1 | Flooding | Very  High | Agaie, Bida, Borgu, Bosso,  Chanchaga, Edati, Gbako, Katcha, Kontagora, Lapai, Lavun, Mariga, Mashegu, Mokwa, Rafi, Rijau, Shiroro,  Suleja, Wushishi | May: Lowest  Jun: Moderate  Jul: High | Aug: Peak  Sep: Peak  Oct: Peak Nov: High  Dec: Moderate  Jan: Lowest | Almost  Certain | Critical | Very High | Low | Good | 18/May/2025 |
| 2 | Banditry/Kidnapping | Very  High | Borgu, Kontagora, Mariga, Mashegu, Muya, Paikoro, Rafi, Shiroro, Wushishi |  |  | Almost  Certain | Severe | Very High | Partial | Good | 18/May/2025 |
| 3 | Boat Mishap | Very  High | Agwara, Borgu, Katcha, Mokwa,  Shiroro, Wushishi | May: Lowest  Jun: Moderate  Jul: High |  | Almost  Certain | Severe | Very High | Partial | Good | 18/May/2025 |
| 4 | Cholera/Acute  Watery Diarrhea | Very  High | Agaie, Agwara, Bida, Bosso,  Chanchaga, Edati, Gurara, Katcha, Kontagora, Lapai, Lavun, Magama, Mariga, Mashegu, Mokwa, Muya,  Paikoro, Rafi, Rijau, Shiroro, Suleja, Tafa, Wushishi | May: High  Jun: Peak  Jul: Peak | Aug: High  Sep: High  Oct: Moderate  Nov: Moderate  Dec: Lowest  Jan: Lowest | Almost  Certain | Severe | Very High | Partial | Good | 17/May/2025 |
| 5 | Deforestation | Very  High | Edati, Lapai, Lavun, Mokwa, Wushishi |  |  | Almost  Certain | Severe | High | Partial | Good | 17/May/2025 |
| 6 | Rain/Wind Storm | Very  High | Agaie, Agwara, Bosso, Chanchaga, Gbako, Kontagora, Lapai, Lavun, Mariga, Mashegu, Mokwa, Paikoro, Rijau, Shiroro, Suleja, Wushishi | May: Peak  Jun: High  Jul: Moderate | Aug: High  Sep: Peak  Oct: Peak  Nov: Moderate  Dec: Lowest  Jan: Lowest | Very Likely | Critical | Very High | Low | Good | 18/May/2025 |
| 7 | Road Traffic Accident | Very  High | Agaie, Bida, Bosso, Chanchaga,  Gurara, Kontagora, Lapai, Mokwa, Suleja |  |  | Almost  Certain | Severe | High | Partial | Good | 18/May/2025 |
| 8 | Fire Outbreak | High | Agaie, Bida, Borgu, Bosso,  Chanchaga, Kontagora, Lapai,  Mokwa, Rafi, Shiroro, Suleja |  |  | Very Likely | Severe | High | Partial | Good | 18/May/2025 |
| 9 | Erosion | High | Agaie, Bosso, Chanchaga, Katcha, Kontagora, Lapai, Mashegu, Mokwa, Tafa |  |  | Almost  Certain | Moderate | High | Partial | Good | 18/May/2025 |
| 10 | Lassa Fever | High | Bida, Suleja, Tafa |  |  | Almost  Certain | Moderate | High | Partial | Good | 17/May/2025 |
| 11 | Measles | High | Agaie, Agwara, Bida, Bosso,  Chanchaga, Edati, Gbako, Gurara, Katcha, Kontagora, Lapai, Lavun, Magama, Mariga, Mashegu, Mokwa,  Muya, Paikoro, Rafi, Rijau, Shiroro, Suleja, Tafa, Wushishi | May: Moderate  Jun: Moderate  Jul: Lowest |  | Almost  Certain | Moderate | High | Partial | Good | 17/May/2025 |
| 12 | Drought | High | Gurara, Kontagora, Magama, Mokwa, Shiroro, Wushishi | May: High  Jun: Peak  Jul: Peak | Aug: High  Sep: High  Oct: High  Nov: Moderate  Dec: Moderate  Jan: Lowest | Very Likely | Moderate | Partial | Low | Unsatisfactory | 17/May/2025 |
| 13 | Substance Abuse | High | Chanchaga, Kontagora, Mariga,  Suleja |  |  | Very Likely | Moderate | High | Partial | Satisfactory | 18/May/2025 |
| 14 | Acute Flaccid  Paralysis | Moderate | Agaie, Agwara, Bida, Bosso,  Chanchaga, Edati, Gbako, Gurara, Katcha, Kontagora, Lapai, Lavun, Magama, Mariga, Mashegu, Mokwa, Muya, Paikoro, Rafi, Rijau, Shiroro,  Suleja, Tafa, Wushishi |  |  | Almost  Certain | Minor | High | High | Good | 17/May/2025 |
| 15 | Food Insecurity | Moderate | Agaie, Agwara, Bosso, Lavun,  Magama, Rafi, Shiroro |  |  | Likely | Moderate | Partial | Partial | Satisfactory | 17/May/2025 |
| 16 | Meningitis | Moderate | Bida, Bosso, Chanchaga, Edati, Gbako, Gurara, Katcha, Kontagora,  Lavun, Magama, Mokwa, Paikoro, Rijau, Suleja, Tafa |  |  | Likely | Moderate | High | Partial | Good | 17/May/2025 |
| 17 | Anthrax | Moderate | Suleja |  |  | Unlikely | Severe | High | Low | Satisfactory | 17/May/2025 |
| 18 | Diphtheria | Low | Bida, Kontagora, Mariga, Suleja, Tafa |  |  | Likely | Minor | Partial | Partial | Good | 17/May/2025 |
